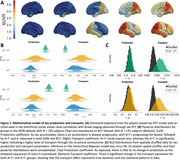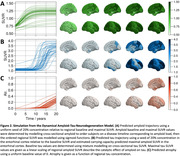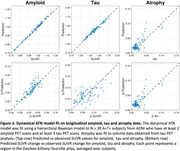# Regional Amyloid Load Predicts Tau and Atrophy Dynamics

**DOI:** 10.1002/alz.085545

**Published:** 2025-01-09

**Authors:** Pavan Chaggar, Jacob W. Vogel, Alexa Pichet Binette, Travis B Thompson, Olof Strandberg, Niklas Mattsson‐Carlgren, Linda Karlsson, Erik Stomrud, Saad Jbabdi, Stefano Magon, Gregory Klein, Oskar Hansson, Alain Goriely

**Affiliations:** ^1^ Oxford University, Oxford, Oxfordshire UK; ^2^ Department of Clinical Sciences Malmö, SciLifeLab, Lund University, Lund Sweden; ^3^ Clinical Memory Research Unit, Lund University, Lund Sweden; ^4^ Clinical Memory Research Unit, Department of Clinical Sciences, Lund University, Lund Sweden; ^5^ Texas Tech University, Texas, TX USA; ^6^ Skåne University Hospital, Lund Sweden; ^7^ Clinical Memory Research Unit, Lund University, Malmö Sweden; ^8^ Wallenberg Center for Molecular Medicine, Lund University, Lund Sweden; ^9^ Memory Clinic, Skåne University Hospital, Malmö Sweden; ^10^ University of Oxford, Oxford, Oxfordshire UK; ^11^ Roche Pharma Research and Early Development, Neuroscience and Rare Diseases Biomarkers, Basel Switzerland; ^12^ Roche Pharma Research and Early Development, FHoffmann‐La RocheLtd, Basel Switzerland; ^13^ Skåne University Hospital, Malmö, 21428 Skåne Sweden; ^14^ University of Oxford, Oxford UK

## Abstract

**Background:**

A generative model of tau PET was applied to multiple cohorts across the Alzheimer's disease (AD) spectrum, revealing longitudinal changes in tau production and transport. A generalisation of the model accounts for amyloid, tau and neurodegeneration (ATN) interactions and accurately explains longitudinal ATN biomarker data, adding potential for region specific and individualized tracking of ATN biomarkers.

**Method:**

A model of tau spreading and production as measured through PET was developed and applied to longitudinal data from amyloid negative (A‐), amyloid positive tau negative (A+T‐) and amyloid positive tau positive (A+T+) cohorts from the Alzheimer’s disease neuroimaging iniative (ADNI; N = 159) and BioFINDER‐2 (BF2; N = 135) datasets. The model was extended to include ATN biomarkers and their interactions, explaining regional tau and atrophy dynamics as a by‐product of regional amyloid load. This model was applied to longitudinal amyloid PET, tau PET and atrophy data from A+T+ subjects in ADNI (N = 39).

**Result:**

The tau PET model faithfully reproduces Braak staging (Figure 1a). In A+T‐ subjects (N = 39 ADNI; N = 18 BF2) inference reveals faster tau spreading through the brain network and slower tau production, compared to A+T+ subjects (N=57 ADNI; N = 54 BF2) who show a higher production rate with slower transport (Figure 1b). This implies greater movement of tau during early AD before an acceleration in production when participants have high levels of both amyloid and tau. This is supported by comparison with spatially shuffled null models (Figure 1c). The generalization to the dynamical ATN model shows that an amyloid‐induced acceleration in tau production explains regional variations in tau load (as measured through PET), Braak staging and downstream neurodegeneration (Figure 2). The ATN model calibrated to longitudinal data from ADNI can accurately describe regional longitudinal ATN biomarkers (Figure 3).

**Conclusion:**

Tau production and transport dynamics vary across the AD spectrum, characterised by an early period of fast tau spreading through the brain network followed by accelerated production of tau aggregates. A dynamical ATN model explains variations in tau and neurodegeneration through regional amyloid load and allows for accurate simulation of personalised ATN biomarkers.